# Immune myocarditis related to sintilimab treatment in a patient with advanced lung adenocarcinoma: A case report

**DOI:** 10.3389/fcvm.2022.955527

**Published:** 2022-10-06

**Authors:** Yunling Lin, Xun Yuan, Lianglong Chen

**Affiliations:** Department of Cardiology, Fujian Medical University Union Hospital, Fujian Cardiovascular Medical Center, Fujian Institute of Coronary Heart Disease, Fuzhou, China

**Keywords:** PD-1, myocarditis, sintilimab, lung adenocarcinoma, cardio-oncology, immune-related adverse events

## Abstract

Immune checkpoint inhibitors (ICI) have improved clinical outcomes of patients with advanced lung cancer, but may lead to fatal cardiac injury. We describe a 66-year-old man with advanced lung adenocarcinoma who presented with chest pain and dyspnea 3 weeks after the first dose of sintilimab. The initial electrocardiogram (ECG) demonstrated ST-elevation in leads V5-V9, and a high-sensitivity troponin level was significantly elevated. However, coronary angiography did not reveal any significant stenosis. The patient was successfully treated with methylprednisolone and immunoglobulin. Cardiac MRI was carried out before discharge and late gadolinium enhancement (LGE) was found to be in the mid layer of the septal segment and the subepicardial layer of the inferolateral wall. Due to the high fatality, ICI-related myocarditis requires close surveillance, prompt management and long-term follow-up.

Data from the National Cancer Center shows that lung cancer is the leading causes of cancer morbidity and mortality in China (1). Monoclonal antibodies targeting inhibitory immune checkpoints (ICI) have significantly improved results for patients with advanced-stage lung cancer (2). However, treatment with ICI is accompanied by immune-related adverse events (irAEs) (3). The immune-related adverse events that most often affect the colon, the liver, the lungs, and the skin, although some rare events may affect the heart as well. Cardiotoxicity induced by ICI may present with arrhythmias, cardiomyopathy, myocarditis, pericarditis and vasculitis. Myocarditis has emerged as an rare, but often fatal complication (4).

Sintilimab has shown positive results in people with relapsed/refractory (RR) Hodgkin's lymphoma, non-small cell lung cancer, esophagealcarcinoma (5–8). We report here a case of a 66-year-old man with advanced lung adenocarcinoma who developed acute myocarditis. The patient was treated successfully with IV methylprednisolone and immunoglobulin. We present the following case in accordance with the CARE reporting checklist.

A 66-year-od man with advanced lung adenocarcinoma was admitted to the local chest pain center with progressive chest pain, dyspnea. He had been diagnosed with advanced lung adenocarcinoma 4 month earlier. He had no history of autoimmune or cardiovascular diseases. Cardiac screening tests including cardiac biomarkers, ECG (Figure 1), and echocardiography showed normal results before chemotherapy. The patient was treated with four cycles of albumin paclitaxel plus carboplatin. The CT scan showed that the patient was not responding to chemotherapy. Then he started albumin paclitaxel plus carboplatin for chemotherapy combined with sintilimab (200 mg/injection). 3 weeks after sintilimab initiation, the patient presented with chest pain, shortness of breath and was admitted to the hospital. His vital signs were unstable on arrival (temperature 36.7°C, heart rate, 84 beats/min; respiratory rate 17 breaths/min, blood pressure 84/52 mmHg. The admission ECG showed sinus rhythm with ST-segment elevation in leads V5 through V9 (Figures 2, 3); laboratory test showed that cardiac troponin-I was 9.4 ng/mL (normal range, 0.00–0.03 ng/mL), CK 922IU/L (normal range, 22-270IU/L), CK-MB 109 IU/L (normal range, 2-25 IU/L);NT-proBNP 8290pg/ml (normal range, 0-349pg/ml). Transthoracic echocardiography showed anterior hypokinaesia, severe left ventricular function impairment with a left ventricular ejection fraction of 35%, mild pericardial effusion (Supplementary Videos 4–7). Coronary angiography was performed showing normal coronary arteries (Supplementary Videos 1–3). A diagnosis of sintilimab associated myocarditis was considered. The patient received methylprednisolone (2 mg/kg) and immunoglobulin (0.4g/kg/d) intravenously for 7 days. At day 4, the ECG showed that the elevated ST-segment had fallen back (Figures 4, 5). At day 8, cardiac magnetic resonance was performed and showed late gadolinium enhancement in the mid layer of the septal segment and the subepicardial layer of the inferolateral wall (Figure 6), with an ejection fraction of 47% (Supplementary Video 11). Gradually, the patient's symptoms improved and his vital signs were stable. The lab tests showed persistent decrease of hs-cTnI, myocardial enzyme indexes (Table 1). Echocardiogram revealed improved left ventricular systolic function, with an ejection fraction of 52% (Supplementary Videos 8–10). The dose of glucocorticoid therapy was gradually reduced. The patient was discharged 10 days after admission. He received oral prednisone tablets with gradually decreasing doses and regular follow-up.

**Figure 1:**
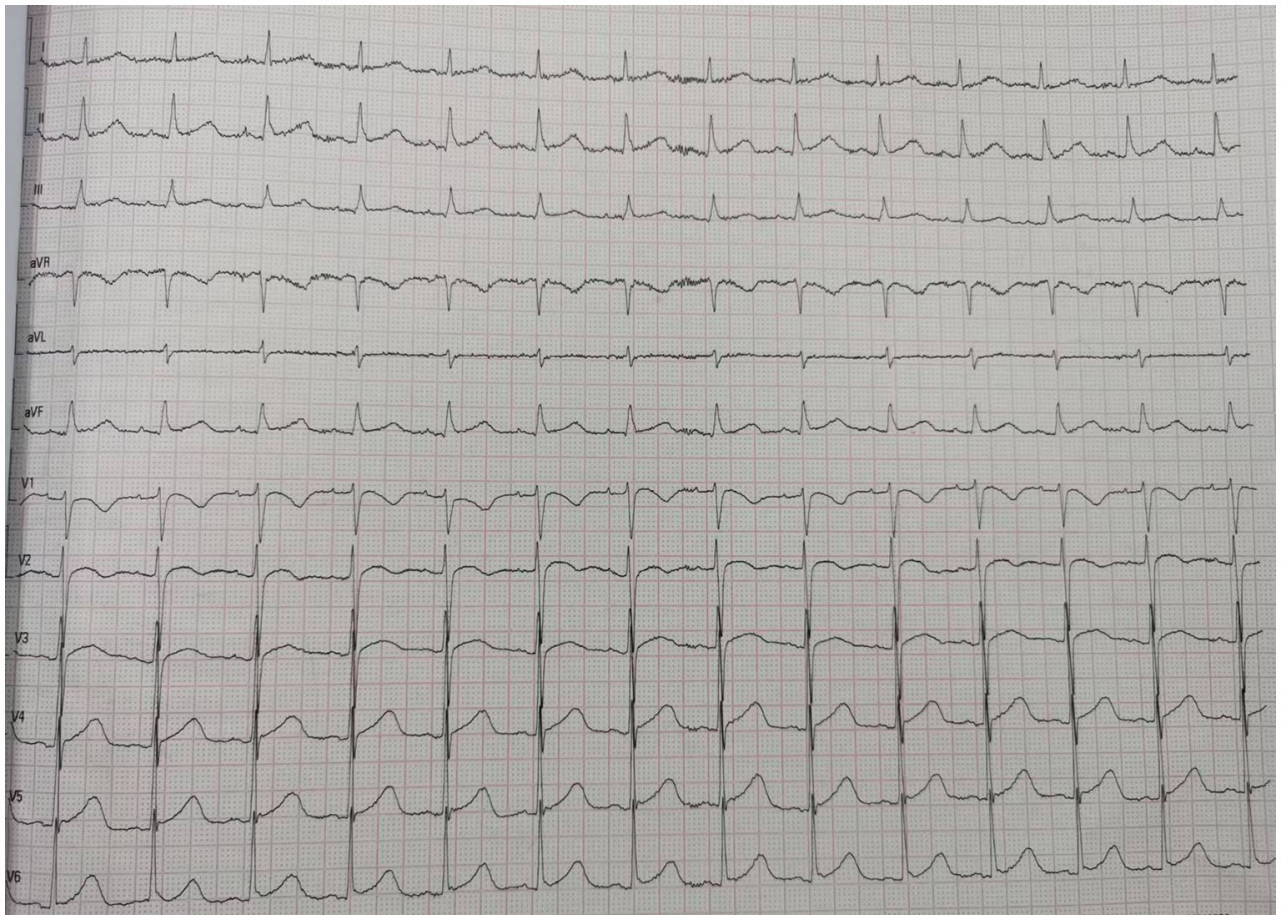
ECG before chemotherapy.

**Figure 2:**
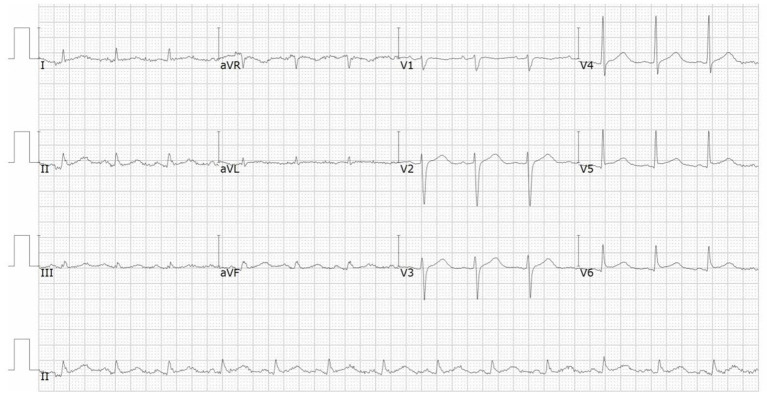
ECG on admission showed ST-segment elevation in leads V5 and V6.

**Figure 3:**
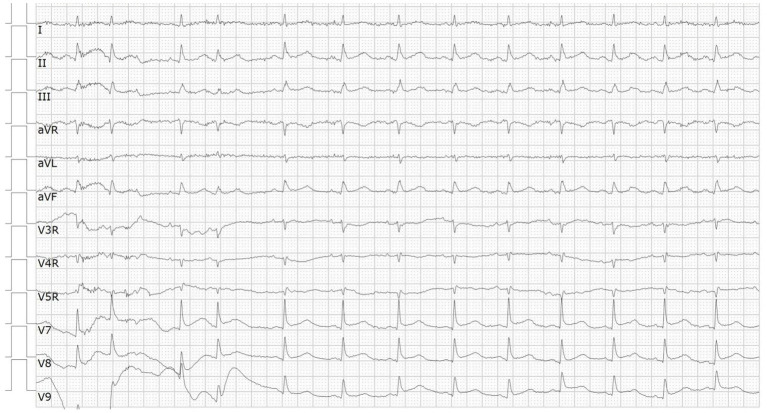
ECG on admission showed ST-segment elevation in leads V7 through V9.

**Figure 4:**
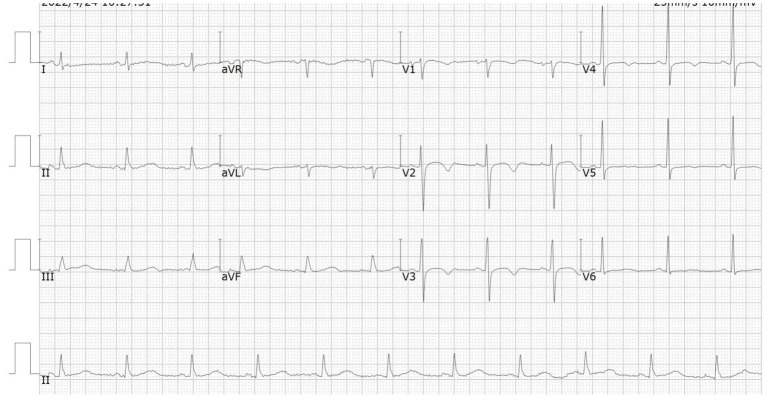
ECG at day 4 showed the elevated ST-segment had fallen back.

**Figure 5:**
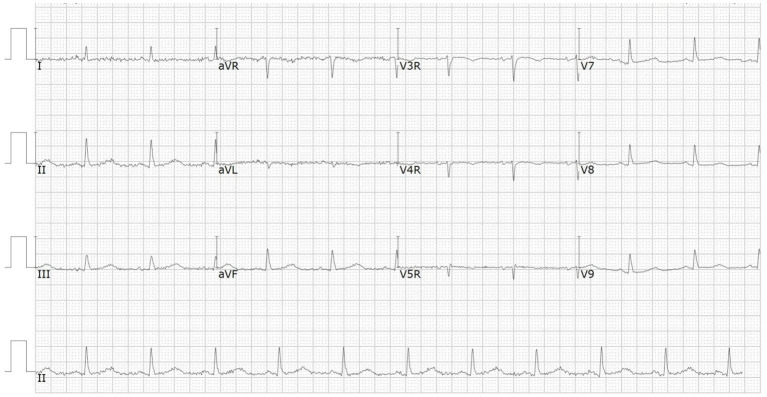
ECG at day 4 showed the elevated ST-segment in leads V7 through V9 had fallen back.

**Figure 6:**
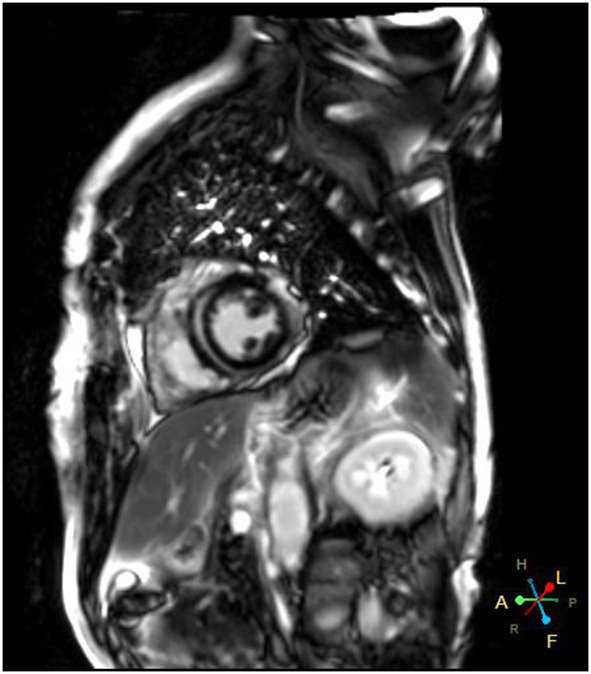
Cardiac magnetic resonance showed late gadolinium enhancement in left ventricular wall.

**Table 1:** The chronological changes of major parameters.

**Days after sintilimab infusion**	**0**	**20**	**30**
LVEF, %	65	35	52
TnI, ng/mL	0.02	9.4	0.42
NT-proBNP, pg/ml	28	8,290	1,265
CKMB, IU/L	13	109	24

CK, creatine kinase; LVEF, left ventricular ejection fraction; NT-proBNP, N-terminal pro-brain natriuretic peptide; hs-TnI, high-sensitivity Troponin I.

## Discussion

In recent years, Immune checkpoint inhibitors has become more widely used in clinical practice and improved the prognosis of patients with malignancy. In the meantime, ICI have been reported to induce cardiovascular toxicities, including myocarditis, pericarditis and vasculitis. The incidence of ICI related myocarditis has ranged from 0.27 to 1.14% (9), but it is often severe and life threatening. The reported incidence of ICIs-related myocarditis is low (<1%), it can be life threatening with a mortality rate up to 60 % (10, 11). Endomyocardial biopsy remains the gold standard for definitive diagnosis of ICI-induced myocarditis but it is not widely used because of its procedural risks and technical limitations. Cardiac magnetic resonance (CMR) has evolved as an standard noninvasive tool for the diagnosis and evaluation of myocarditis. Late gadolinium enhancement (LGE) is associated with a poor prognosis in patients with ICI-induced myocarditis (12). Myocardial biopsy and CMR are often not available in the setting of an acute presentation. It has been demonstrated that 2D/3D speckle-tracking strain imaging echocardiography may be promising diagnostic and prognostic tool in acute myocarditis, even in patients with preserved left-ventricular ejection fraction (13). However, the major pitfalls of speckle-tracking are its dependency on image quality and its ability to define the endocardial and epicardial boundaries. Once diagnosed, treatment should be initiated immediately. However, due to the absence of high-level evidence studies, there are still no standard therapeutic strategies. Glucocorticoid treatment is recommended for ICI-associated myocarditis but the effective dosage and optimal duration of corticosteroid therapy are not precisely recommended. Due to limited clinical data, the utility of other immunosuppressive agents is not well established.

Sintilimab is a humanized monoclonal IgG4 antibody which was approved for the treatment of hematological cancers and several advanced solid tumors in China. The epitope of the sintilimab/PD-1 complex is located at the FG loop of PD-1, which is different from that of nivolumab or pembrolizumab Compared to nivolumab or pembrolizumab, sintilimab has a higher binding affinity with more PD-1 molecules on CD3+T cells, and has better T cell activating characteristics. Sintilimab exhibited good safety and tolerability in clinical studies (5–8). Compared to nivolumab or pembrolizumab, sintilimab has a higher binding affinity with more PD-1 molecules on CD3+T cells, and has better T cell activating characteristics. For sintilimab, the incidence of fatal treatment-related adverse events (TRAEs) ranges from 1 to 6.25%. The incidence of TRAEs for sintilimab monotherapy ranges from 0 to 2.5%. It seems to be higher than average (14). Four cases of sintilimab-induced myocarditis have been reported recently (15–18), but cardiac magnetic resonance imaging was not performed in all of these patients. In our case, the echocardiography showed that cardiac function was severely damaged with a LVEF of 35% on admission. A significant improvement of left ventricular systolic function with a LVEF of 52% was noted several days after treatment. However, cardiac MRI showed extensive myocardium fibrosis. Therefore, longer follow-up is warranted to determine whether myocardial fibrosis can fully regress and to observe the the long-term prognosis of the patient.

## Data availability statement

The raw data supporting the conclusions of this article will be made available by the authors, without undue reservation.

## Ethics statement

Written informed consent was obtained from the participant for the publication of this case report. Written informed consent was obtained from the individual(s) for the publication of any potentially identifiable images or data included in this article.

## Author contributions

YL: contributed to the clinical care of the patient data analysis, writing of the article, and editing of the figure. XY: contributed to the literature search and clinical record collection. LC: contributed to study design and critical review of the article. All authors contributed to the article and approved the submitted version.

## Funding

This study was financially supported by the funding for Top Hospital and Specialty Excellence of Fujian Province (grant no. 212790530102) and the Provincial Natural Science Foundation of Fujian (grant no. 2022J01253), China.

## Conflict of interest

The authors declare that the research was conducted in the absence of any commercial or financial relationships that could be construed as a potential conflict of interest.

## Publisher's note

All claims expressed in this article are solely those of the authors and do not necessarily represent those of their affiliated organizations, or those of the publisher, the editors and the reviewers. Any product that may be evaluated in this article, or claim that may be made by its manufacturer, is not guaranteed or endorsed by the publisher.
